# Evaluation of cognitive impairment in elderly population with hypertension from a low-resource setting: Agreement and bias between screening tools

**DOI:** 10.1016/j.ensci.2016.11.012

**Published:** 2016-12-01

**Authors:** María Lazo-Porras, María A. Pesantes, J. Jaime Miranda, Antonio Bernabe-Ortiz

**Affiliations:** a CRONICAS Center of Excellence in Chronic Diseases, Universidad Peruana Cayetano Heredia, Lima, Peru; b CONEVID Unidad de Conocimiento y Evidencia, Universidad Peruana Cayetano Heredia, Lima, Peru; c School of Medicine, Universidad Peruana Cayetano Heredia, Lima, Peru; d Faculty of Epidemiology and Population Health, London School of Hygiene and Tropical Medicine, London, United Kingdom

**Keywords:** Cognitive impairment, Hypertension, Minimental state examination, Montreal cognitive assessment, Leganés cognitive test

## Abstract

**Introduction:**

The evaluation of cognitive impairment in adulthood merits attention in societies in transition and especially in people with chronic diseases. Screening tools available for clinical practice and epidemiological studies have been designed in high-income but not in resource-constrained settings. The aim of this study was to assess the agreement and bias of three common tools used for screening of cognitive impairment in people with hypertension: the modified Mini-Mental State Examination (MMSE), the Montreal Cognitive Assessment (MoCA), and the Leganés Cognitive Test (LCT).

**Methods:**

A cross-sectional study enrolling participants with hypertension from a semi-urban area in Peru was performed. The three screening tools for cognitive impairment were applied on three consecutive days. The prevalence of cognitive impairment was calculated for each test. Pearson's correlation coefficients, Bland-Altman plots, and Kappa statistics were used to assess agreement and bias between screening tools.

**Results:**

We evaluated 139 participants, mean age 76.5 years (SD ± 6.9), 56.1% females. Cognitive impairment was found in 28.1% of individuals using LCT, 63.3% using MMSE, and 100% using MoCA. Correlation coefficients ranged from 0.501 between LCT and MoCA, to 0.698 between MMSE and MoCA. Bland-Altman plots confirmed bias between screening tests. The agreement between MMSE and LCT was 60.4%, between MMSE and MoCA was 63.3%, and between MoCA and LCT was 28.1%.

**Conclusions:**

Three of the most commonly used screening tests to evaluate cognitive impairment showed major discrepancies in a resource-constrained setting, signaling towards a sorely need to develop and validate appropriate tools.

## Highlights

• Cognitive impairment in elderly with hypertension can range between 28% and 100% according to the screening tool utilized.
• Three of the most common tools to evaluate cognitive impairment have poor agreement in older people with hypertension.
• The agreement between cognitive impairment tests is lower among persons with greater scores.

## 1 Introduction

Cognitive impairment is a growing public health concern [1], [2], [3]. Aging, lifestyles and chronic diseases, mainly hypertension and type 2 diabetes mellitus, are the most important contributing factors for the development of and progression towards cognitive impairment [4]. Previous studies in Latin America have reported a prevalence of cognitive impairment between 1% to 28% in the general population [5], [6], whereas dementia was present in 3.4% to 7.1% [6], [7]. The wide range reported for these estimates depend on the method used, i.e. screening tools or specialized clinical assessment. Within the arsenal of screening tools, several tests including the modified Minimental State Examination (MMSE), Leganés Cognitive Test (LCT), Montreal Cognitive Assessment (MoCA), and others have been validated against international guidelines or clinical plus an assessment battery for cognitive impairment diagnosis.

Even when international guidelines [8], [9] recommend older adults (i.e. those aged ≥ 65 years) should be assessed for cognitive impairment, yet a definite diagnosis of cognitive impairment is cumbersome and must be performed by a neurologist [10], [11]. However, other guidelines and researchers concluded that the evidence is insufficient to assess the balance of harms and benefits of the screening [12], [13]. Therefore, utility of cognitive screening is still controversial. Moreover, there are reported different strategies for screening i.e. use of one test, two test in combination and nowadays there is not agreement in the best method of evaluation [14]. Another issue is that several older adults, especially in low- and middle-income countries, live in remote rural regions or urban areas without access to specialized services that could offer routine screenings; hence, the need to have simple and rapid screening tools to assess cognitive impairment.

Most of the epidemiological studies using different tests for cognitive impairment and dementia have been developed and validated in high-income countries where language, socioeconomic status, education level, and access to healthcare are different from Latin America. The literature on the topic arising from Latin America is scant, coming mainly from Brazil. For example, there are studies adapting and validating the MMSE in Brazil, but also studying the effect of age and education on results, as well as assessed different cut-offs [15], [16], [17]. Moreover, a previous study used the MMSE in a rural population with low education [18]. In addition, MoCA has been also validated in Brazil [19]. In Ecuador some studies have used LCT and MoCA [20], [21]. Despite of this, there is limited data available evaluating the performance of these screening tools to diagnose or suggest cognitive impairment in Latin American and its rural areas [22].

The aim of this study was to determine the bias and agreement between the MMSE, MoCA and LCT for screening of cognitive impairment among participants with hypertension in a semi-urban area in Peru, and to provide evidence about the need to have more appropriate tools given current changes in the population trends and recent efforts by the Peruvian government to improve the quality of life among poor older adults [23].

## 2 Methods

### 2.1 Ethics

The Institutional Review Board of the Universidad Peruana Cayetano Heredia, Lima, Peru, approved this protocol. Oral informed consent was obtained from all participants due to high rates of illiteracy.

### 2.2 Study design, setting and participants

This study was performed in the semi-urban area of Tumbes, located in the northern coast of Peru, near the border with Ecuador, where the traditional agricultural landscape has become intermixed with rapidly growing urban sections. As people with hypertension are at higher risk of cognitive impairment, participants were a sub-sample of those originally enrolled in the Tumbes site of the CRONICAS Cohort Study [24], specifically those aged ≥ 65 years and classified as having a diagnosis of hypertension at baseline in 2010–2011. Hypertension was defined according to international guidelines: systolic blood pressure ≥ 140 mm Hg or diastolic blood pressure ≥ 90 mm Hg using the mean of the last two of three blood pressure (BP) measures, or self-report of physician diagnosis and currently receiving antihypertensive medication [25]. All participants who met the inclusion criteria were re-contacted during October and November of 2014 to be assessed with the screening tools for cognitive impairment.

### 2.3 Cognitive evaluation

Three different screening tools were used; each has been previously translated into Spanish and used in different Spanish-speaking countries. Our evaluation was conducted during three consecutive days. Every day, a trained staff performed one of the tests in the following order: MMSE was applied on the first day, followed by the Leganés on the second day, and the MoCA on the last day. This method was used to avoid fatigue given the similarity of questions across tools as well as to ensure a standardized procedure for the evaluation of the participants.

The modified Minimental State Examination (MMSE) was validated in Spanish in Chile, contains six questions with a maximum score of 19 points. A cutoff of 13 points or lower suggests cognitive impairment [26], [27]. Items assessed in the tool are registration, orientation, delayed recall, attention/concentration, visual-spacial ability and verbal comprehension.

The Montreal Cognitive Assessment (MoCA), validated in Colombia [28], is a 30-point assessment tool comprising 11 questions. According to the manual we added one point for an individual who has 12 years or fewer of formal education. A cutoff ≤ 25 points indicates cognitive impairment [29]. Items evaluated in this tool are attention and concentration, executive functions, memory, language, conceptual thinking, calculation, visuoconstructional skills, and orientation.

The Leganés Cognitive Test (LCT) was validated in Spanish in Spain [30]. The tool contains 12 questions with a maximum score of 32 points, and a cutoff point of 22 is used for determining cognitive impairment [31]. Items included in this tool are temporal orientation, spatial orientation, personal information, naming test, immediate memory, late memory and logical memory.

### 2.4 Other variables

We collected demographic information: age, sex, education level (none/initial, primary, secondary or higher), marital status (single, with partner, divorce/widowed), currently working, socioeconomic status based on possessions weighted asset index, and split in tertiles. With regards to hypertension status, we collected time of disease in years, antihypertensive treatment, and control of blood pressure defined as systolic blood pressure < 140 mg/dL and diastolic blood pressure < 90 mm Hg.

Lifestyles variables included smoking status (never, former, current smoker), hazardous drinking, evaluated using the Alcohol Use Disorder Identification Test [32], physical activity using the leisure time and transport-related domain of the International Physical Activity Questionnaire (IPAQ) [33], [34], and access to healthcare (yes/no).

Other important clinical variables such as the presence of depressive symptoms using the Center for Epidemiological Studies Depression (CES-D) with a cutoff of 16 points; type 2 diabetes mellitus, defined as fasting glucose ≥ 126 mg/dL [≥ 7 mmol/L] or self-report of physician diagnosis and currently receiving anti-diabetic medication [35]; current self-reported history of stroke, body mass index (BMI) categories (normal if BMI ≥ 18.5 and < 25 kg/m^2^, overweight ≥ 25 and < 30 kg/m^2^, and obese ≥ 30 kg/m^2^) [36], were also evaluated.

### 2.5 Statistical analysis

STATA 12 for Windows (Stata Corp, College Station, TX) was used for analysis. Prevalence of cognitive impairment using MMSE, MoCA and LCT and their respective 95% confidence intervals (95% CI) were estimated. Mean and standard deviation (SD) or median and interquartile range (IQR) were utilized to describe the distribution of quantitative variables. To allow for comparisons between tests, we used non-standardized (raw scores) and standardized (z-score) values of MMSE, MoCA and LCT. This standardization technique rescaled raw scores into a new variable with a mean of 0 and a standard deviation of 1. Pearson correlation coefficients (r) were calculated to measure the strength of the linear relationship between two tests. As previous studies comparing two quantitative methods using correlation have been criticized, Bland-Altman plots, calculated using the difference between the methods (X − Y) against the average of them (X + Y) / 2 [37], were used. This simple parametric approach allows us to assess error and bias, spot outliers and detect trends.

Finally, using each of the score's cutoff values for cognitive impairment, Kappa statistics (κ) were calculated.

## 3 Results

### 3.1 Description of participants

From the 1160 participants recruited in the CRONICAS Cohort Study, a total of 146 had the diagnosis of hypertension and were aged ≥ 65 years old. Of them, 143 were re-contacted, but 4 (2.8%) were excluded due to their visual impairment (blindness). Detailed information regarding selection of participants is shown in Online E-Fig. 1. Therefore, only 139 were assessed for cognitive impairment, their mean age was 76.5 years (SD 6.9) and 56.1% were females. A total of 74/139 (53.6%) lived with a partner, 108/139 (77.7%) had only primary education level, 44/139 (31.7%) still worked. Sociodemographic and lifestyle characteristics are show in Table 1.

**Table 1:** Characteristics of participants with hypertension.

	N = 139
Sociodemographic variables	
Age, mean (SD)	76.5 (6.9)
Female, n (%)	78 (56.1)

Marital status, n (%)	
Single	8 (5.8)
With partner	74 (53.6)
Divorce/widowed	7 (40.6)

Individual's education level, n (%)	
Illiterate	18 (12.9)
Primary	108 (77.7)
Secondary	13 (9.4)

Possessions weighted asset index, n (%)	
Lowest tertile	5 (46.8)
Middle	50 (35.9)
Highest tertile	24 (17.3)

Currently working, n (%)	
Yes	44 (31.7)

Smoking, n (%)	
Never smoke	85 (61.1)
Former smoker	45 (32.4)
Current smoker	9 (6.5)

Hazardous drinking, n (%)	
Yes	5 (3.6)

Leisure-time physical activity, n (%)	
Low	134 (96.4)
Moderate/high	5 (3.6)

Transport-related physical activity, n (%)	
Low	110 (79.1)
Moderate/high	29 (20.9)

Access to healthcare	
Yes	109 (80.2)
SD = standard deviation	

Subjects had a median time of hypertension of 8.3 years (IQR: 5.5–13.8), 80/139 (58.7%) reported currently receiving antihypertensive medication, and 55/139 (39.6%) had their blood pressure under control. Other comorbidities such as depression, type 2 diabetes mellitus, and history of stroke are show in Table 2.

**Table 2:** Clinical variables of study population.

	N = 139
Depressive symptoms, n (%)	
Yes	26 (18.7)

Type 2 diabetes mellitus, n (%)	
Yes	22 (15.8)

Stroke, n (%)	
Yes	1 (0.7)

Body mass index, n (%)	
Normal	43 (30.9)
Overweight	59 (42.5)
Obese	37 (26.6)

Antihypertensive treatment, n (%)	
Yes	80 (57.6)

Control blood pressure, n (%)	
Yes	55 (39.6)

### 3.2 Cognitive evaluation

Cognitive impairment was present in 63.3% of individuals (mean score: 12.4, SD 3.8) when using the MMSE; in 28.1% (mean score: 23.9, SD 3.6) when using the LCT; and in 100% (mean score 14. 8, SD 4.5) when using the MoCA.

**Fig. 1:**
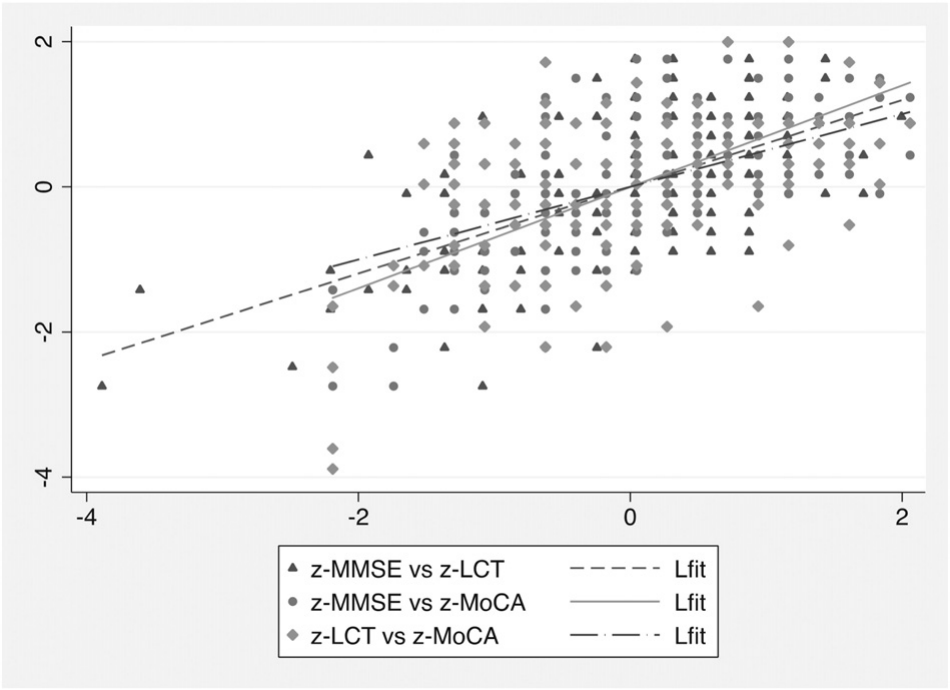
Scatter plots of cognitive impairment tools. ▲ z-modified Minimental State Examination (axial x) and z-Leganés Cognitive Test (axial y). ○ z-modified Minimental State Examination (axial x) and z-Montreal Cognitive Assessment (axial y). ♦ z-Leganés Cognitive Test (axial x) and z-Montreal Cognitive Assessment (axial y).

The correlation between z-MMSE and z-LCT was 0.597 (p < 0.001), between z-MMSE and z-MoCA was 0.698 (p < 0.001), and between z-LCT and z-MoCA was 0.501 (p < 0.001). A scatter plot of standardized scores correlations is shown in Fig. 1, whereas Online E-Fig. 2 shows scatter plots using raw scores.

### 3.3 Bias and agreement between tools

**Fig. 2:**
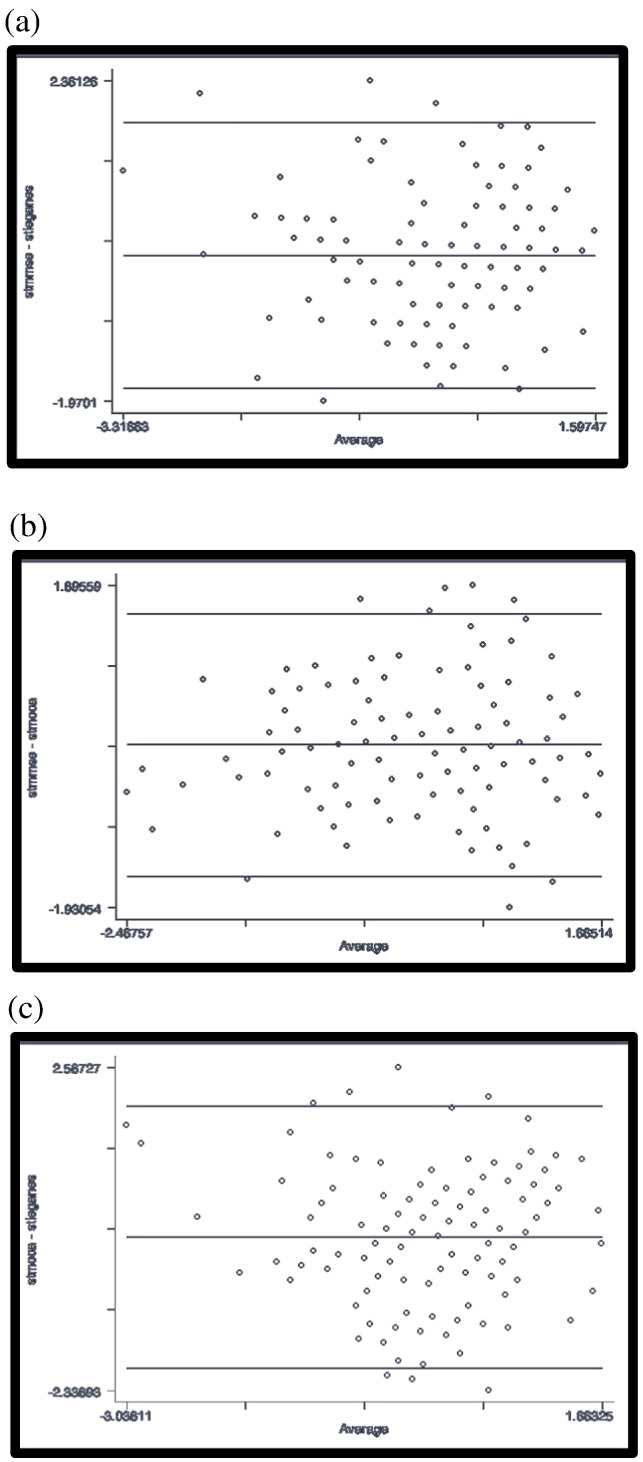
Bland-Altman plots of cognitive impairment tools. Bland-Altman plots of (a) z-MMSE and z-LCT, (b) z-MMSE and z-MoCA and (c) z-MoCA and z-LCT. Central horizontal line represents mean difference between the two tools. Upper and lower solid lines represent the upper and lower limits of agreement of the comparison between tools.

Bland-Altman plots confirmed bias between the tools. The z-MMSE and z-LCT (Fig. 2a) had a limit of agreement from − 1.795 to 1.795. The z-MMSE and z-MoCA (Fig. 2b) had a limit of agreement from − 1.553 to 1.553. Finally, z-LCT and z-MoCA had a limit of agreement from − 1.999 to − 1.999. Online E-Fig. 3 presents Bland-Altman plots using raw scores.

Agreement, as per κ statistic, between the MMSE and LCT was 60.4%, between MMSE and MoCA was 63.3%, and between MoCA and LCT was 28.1%.

## 4 Discussion

### 4.1 Main findings

We used three different well-established screening tools for cognitive impairment in elderly participants with hypertension, low educational level, and low socioeconomic status from a semi-urban area in Peru and found large discrepancies in the rates of cognitive impairment: one third of subjects were classified as having cognitive impairment with the LCT, two thirds with the MMSE, and all of them with the MoCA. Despite of the correlation observed between tools, bias according to Bland-Altman plots showed that for greater levels of cognitive impairment the spread of points around the mean also increased, suggesting that the agreement between tests is lower in persons with greater scores of cognitive impairment, even when most of the points are within one standard deviation. As a result, all the tools evaluated showed diverse results and, as such, these observations call for a major need to initiate work towards finding an appropriate tool or appropriate cutoff point for cognitive impairment screening in low-resource settings where the population has similar characteristics to the ones mentioned: low level of education and income. Anticipating a rise in the burden related to cognitive impairment in economies in transition together with a well-known problem of shortage of specialized human resources, this study signals towards a major gap and challenge for the years to come.

### 4.2 Comparison with other studies

The different domains evaluated by each test might explain the dissimilar results found in our study. For example, whereas the three tests evaluated orientation, MMSE and MoCA evaluated attention and visual-spatial ability, MMSE and LCT evaluated registration and delayed recall, and MoCA and LCT evaluated memory. Prior studies have reported that MoCA has greater sensitivity and specificity compared to the MMSE for screening of cognitive impairment [38]. On the other hand, however, the LCT is a tool easy to administer, and, as the MoCA, is less susceptible to educational level, perhaps because the MMSE includes calculation and drawing. Another important point is that we applied the abbreviated version of the MMSE, even when the original instrument had been developed for low-educated population, the validation is from Chile, who has a population with higher levels of education than Peru [27].

There is scarce information regarding the comparison of the performance of different cognitive impairment tools, especially in low- and middle-income countries or rural settings. For example, a study from Brazil enrolling low-educated elderly individuals found that 11.8% had cognitive impairment using LCT and 37.2% using MMSE, and a poor agreement using Kappa was reported between tests [39]. Similarly, another study in United States used Bland-Altman plots to assess the agreement between Alzheimer's Disease Assessment Scale – Cognitive (ADAS-Cog), Clinical Dementia Rating (CDR), and the MMSE and found that poor agreement between tests was a function of increasing cognitive impairment [40] as our results also suggest.

### 4.3 Public health relevance

As the world population ages, including large populations in low- and middle-income countries, the probability of having cognitive impairment also increases. Cognitive impairment translates into individuals who require special care both at home and by state services.

Even when in last years the screening of cognitive impairment, especially in elderly, is controversial [12], screening is still apply and currently, most health systems face major shortages of human resources, and more specialists in the fields of neurology are needed [41]. Although our study has not been performed to support conclusions about the performance of any screening tool or to identify which one was better, our findings suggest the potential misclassification of individuals with a condition of cognitive impairment with further impact in clinical practice, disease burden, and policy implications, at least in people with hypertension. Apparently, for a low-income scenario as in the case of this study, the LCT is less susceptible to education level in comparison to MMSE, as suggested by literature [30]. In the case of MMSE, previous studies have found that age and education could explain 12% of the variability in results [42].

Tests for cognitive impairment should have the following features: easily administered, promptness of results, and be performed by any health professional or even community health workers. However, once a case is detected using any of the screening tools, a battery of other evaluations, laboratory, and imaging tests are needed to confirm the diagnosis of cognitive impairment. For this reason, a test with higher specificity than sensitivity would be favored as there is no effective treatment for cognitive impairment, but some preventive measures can be taken to delay progress. As a result, false-positive cases would be reduced, together with lesser burden and costs for the patient and its family.

### 4.4 Strengths and limitations

This study assessed three cognitive impairment tools in a resource-constrained setting in Peru. As these tools have been generated and validated for individuals in high-income settings, studies from other settings, including Latin America, are needed to account for appropriate cultural and income validation. This is one of the few studies applied in Spanish speakers from a semi-urban population in Latin America.

Nonetheless, this study has some limitations. First, the findings of this report cannot be generalized to other settings, as the particular characteristics of the study population — presence of hypertension, low educational level, and low socioeconomic status — might partially explain differences. Our sample included only participants with hypertension reducing the external validity of the findings and limited the approach of providing evidence about the need of more adequate instruments. Yet, our results can well serve as a unique opportunity to explore the functioning of such tools in difficult fieldwork circumstances. Second, we did not assess the performance of these screening tools by comparing them with a gold standard test, and therefore we could not estimate sensitivity and specificity estimates neither choose the better tool to study cognitive impairment in future studies. Third, we used cut-off points validated to other populations with different characteristics to our sample. Even when this partially explains the low rate of agreement, these cut-off points are used in clinical practice in most countries of Spanish language [43]. Therefore, physicians could classify a patient as cognitive impaired depending upon the tool utilized. Fourth, we did not use the original MMSE test but the abbreviated version of the MMSE validated in Chile [27], [44]. As this short version does not include abstract thinking, it is appealing for low educated population, although comparisons need to be done with caution. Finally, the tests were applied to participants in the same order instead of random order with the purpose of standardizing the assessment of the participants.

## 5 Conclusions

This study shows that three of the most common tools available to assess cognitive impairment in the elderly population have poor agreement in resource-constrained settings in a Latin America country. This calls for attention in two different ways, first, our results support the controversy of screening cognitive impairment and, second, the need of finding the more appropriate screening tool for cognitive impairment in low-resource settings.

## Financial disclosure

The establishment of the CRONICAS Centre of Excellence in Chronic Diseases at Universidad Peruana Cayetano Heredia was funded in whole with Federal funds from the National Heart, Lung and Blood Institute, National Institutes of Health, Department of Health and Human Services, under Contract Number HHSN268200900033C.

JJM currently receives, or has received during the planning of this study, further support from the Alliance for Health Policy and Systems Research (HQHSR1206660), Consejo Nacional de Ciencia y Tecnología (CONCYTEC), DFID/MRC/Wellcome Global Health Trials (MR/M007405/1), Fogarty International Center (R21TW009982), Grand Challenges Canada (0335-04), the International Development Research Center Canada (106887, 108167), the Inter-American Institute for Global Change Research (IAI CRN3036), the National Heart, Lung and Blood Institute (5U01HL114180, HHSN268200900028C), the National Institute of Mental Health (1U19MH098780), and the Swiss National Science Foundation (40P740-160366). AB-O is a research training fellow in public health and tropical medicine funded by the Wellcome Trust (103994/Z/14/Z). MAP is supported by a postdoctoral fellowship (2014–2016) of the Peruvian National Council for Science and Technology (Consejo Nacional de Ciencia y Tecnología -CONCYTEC).

## Contribution to authorship

ABO conceived and designed the study. MAP was responsible for the fieldwork. MLP and ABO contributed to the data analysis. MLP and ABO drafted the first version of the manuscript with important input from MAP and JJM. All of the authors contributed to the revising of the manuscript and gave their final approval of the version submitted for publication.

## Conflict of interests

None to declare for the authors.

## Appendix A Supplementary data

Online E-Figures Image 1
