# Supplementary material for: Evaluation of cognitive impairment in elderly population with hypertension from a low-resource setting: Agreement and bias between screening tools
Source: eNeurologicalSci. 2016 Dec 1;5:35–40. doi: 10.1016/j.ensci.2016.11.012 (PMC5312667; doi:10.1016/j.ensci.2016.11.012)
Supplement: Supplementary file 1 — Online E-Figures [file mmc1.docx]

**ONLINE SUPPLEMENT**

E. Figure 1. Flowchart of selection of participants


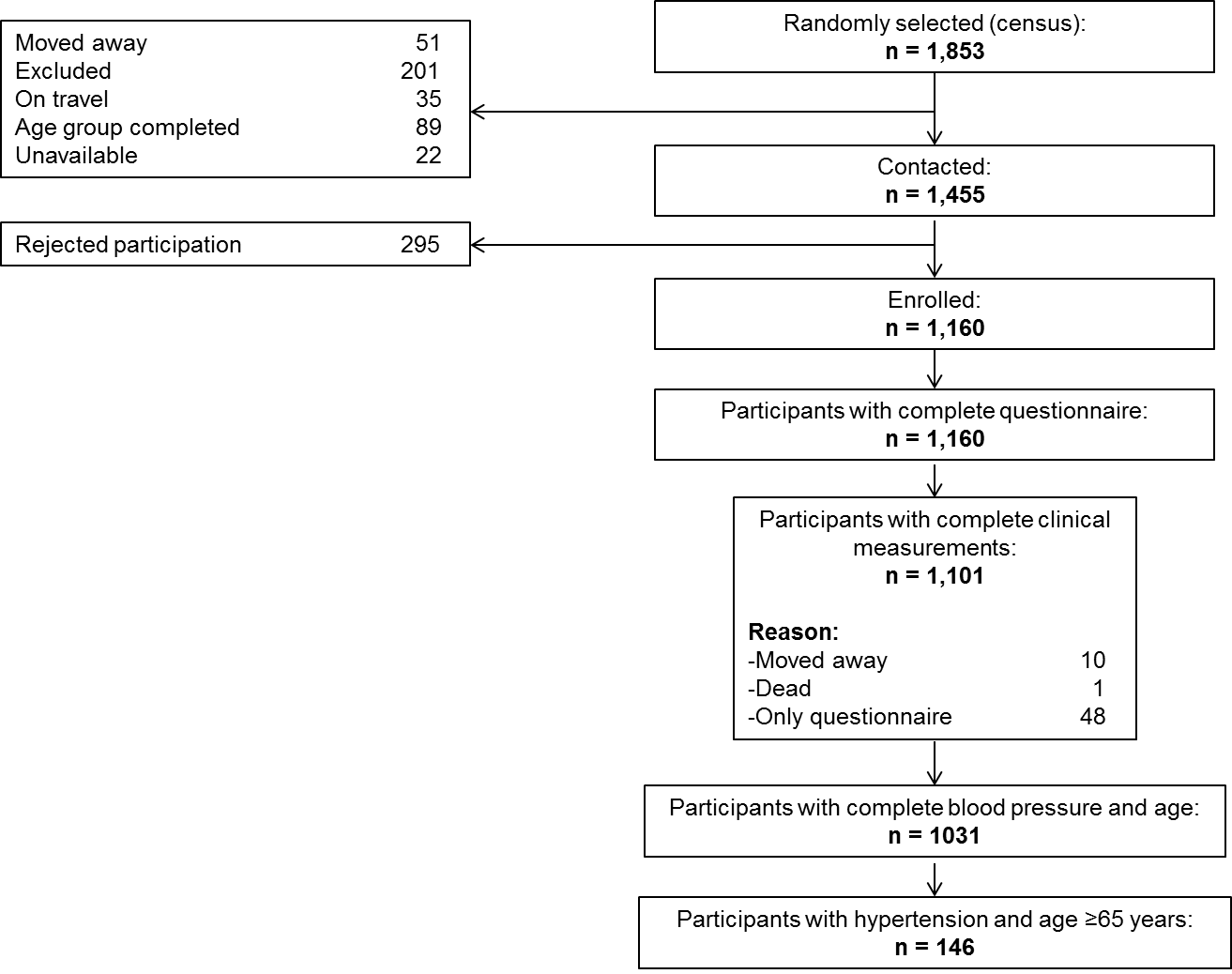


## E-Figure 2. Scatter Plots

modified Minimental State Examination (axial x) and Leganés cognitive test (axial y)

🞆 modified Minimental State Examination (axial x) and Montreal Cognitive Assessment (axial y)

◆ Leganés cognitive test (axial x) and Montreal Cognitive Assessment (axial y)

## E-Figure 3. Bland-Altman plots

Bland-Altman plots of the tools to evaluate cognitive impairment (a) MMSE and LCT (b) MMSE and MoCA. (c) LTC and MoCA. Central horizontal line represents mean difference between the two tools. Upper and lower solid lines represent the upper and lower limits of agreement of the comparison between tools.

(a)


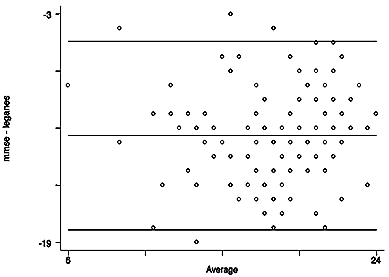


| Limits of agreement (Reference Range for difference): -18.118 to -4.918 |
| --- |
| Mean difference: -11.518 (95%CI -12.071 to -10.965) |

(b)


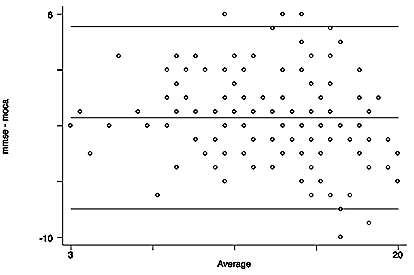


| Limits of agreement (Reference Range for difference): -7.964 to 5.101 |
| --- |
| Mean difference: -1.432 (95%CI -1.979 to -0.884) |

(c)


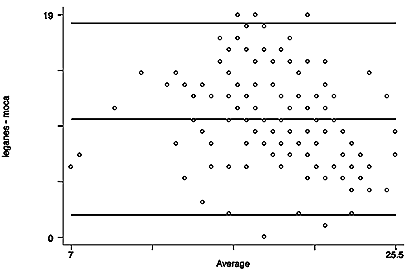


| Limits of agreement (Reference Range for difference): 1.898 to 18.274 |
| --- |
| Mean difference: 10.086 (95%CI 9.400 to 10.773) |
